# High-velocity trauma by social media: a new mechanism of injury in pediatric traumatology?

**DOI:** 10.1007/s00068-026-03361-8

**Published:** 2026-10-19

**Authors:** Michael Sennert, Benjamin Schoof, Merle Körner, Monika Le, Jan Kolman, Omid Madadi-Sanjani, Kristofer Wintges

**Affiliations:** 1 Department of Pediatric Surgery, University Medical Center Hamburg-Eppendorf (UKE), Martinistraße 52, 20246 Hamburg, Germany; 2 Department of Trauma and Orthopedic Surgery, University Medical Center Hamburg-Eppendorf, Martinistraße 52, 20246 Hamburg, Germany

**Keywords:** Social media challenges, Pediatric trauma, Limb injuries, High-energy trauma, Digital behavior

## Abstract

**Purpose:**

Over the past two decades, social media challenges have emerged as a significant cause of injuries among adolescents. This study evaluates a sudden cluster of severe upper limb injuries resulting from the viral “Superman challenge” to raise clinical awareness of digital behaviors in pediatric trauma.

**Methods:**

A retrospective study at a tertiary pediatric trauma center evaluated patients aged < 18 years admitted in December 2024 following the “Superman challenge.”

**Results:**

Eight patients were admitted within a 10-day window following high-energy injuries from the challenge. The cohort showed a female predominance (62.5%) with a mean age of 13.25 (±1.03) years. The high-velocity mechanism led to atypical, severe injury patterns distinct from routine accidental falls. We observed an unusual cluster of severe distal forearm fractures in five patients, which uniformly required surgical treatment. Three patients even suffered bilateral distal radius fractures. Additional associated injuries included fractures of the ulnar styloid process (*n* = 5) and concomitant TFCC tears (*n* = 2), reflecting the high-energy nature of the challenge.

**Conclusions:**

The rapid, viral spread of physical social media challenges such as the ‘Superman Challenge’ can lead to dangerous injury mechanisms involving high-velocity trauma among children and adolescents. These trends could lead to sudden, localized clusters of atypical high-energy injuries—a scenario that, under specific conditions, risks overtaxing conventional healthcare systems, as previously demonstrated in the literature. Medical staff need to be aware of changing digital behaviors, which represent a new mechanism of injury in pediatric trauma.

## Introduction

Fractures of the upper limbs in adolescents are commonly caused by accidental falls, sports-related trauma, and motor vehicle accidents [1]. However, over the past two decades, a significant new mechanism of injury (MOI) has emerged: the rapid viral spread of risky social media challenges (SMCs) [2]. These challenges are defined as activities performed as digital content, often involving a peer-driven “invitation” to join, which carry a reasonable likelihood of causing injury or even death [3–5].

**Fig. 1:**
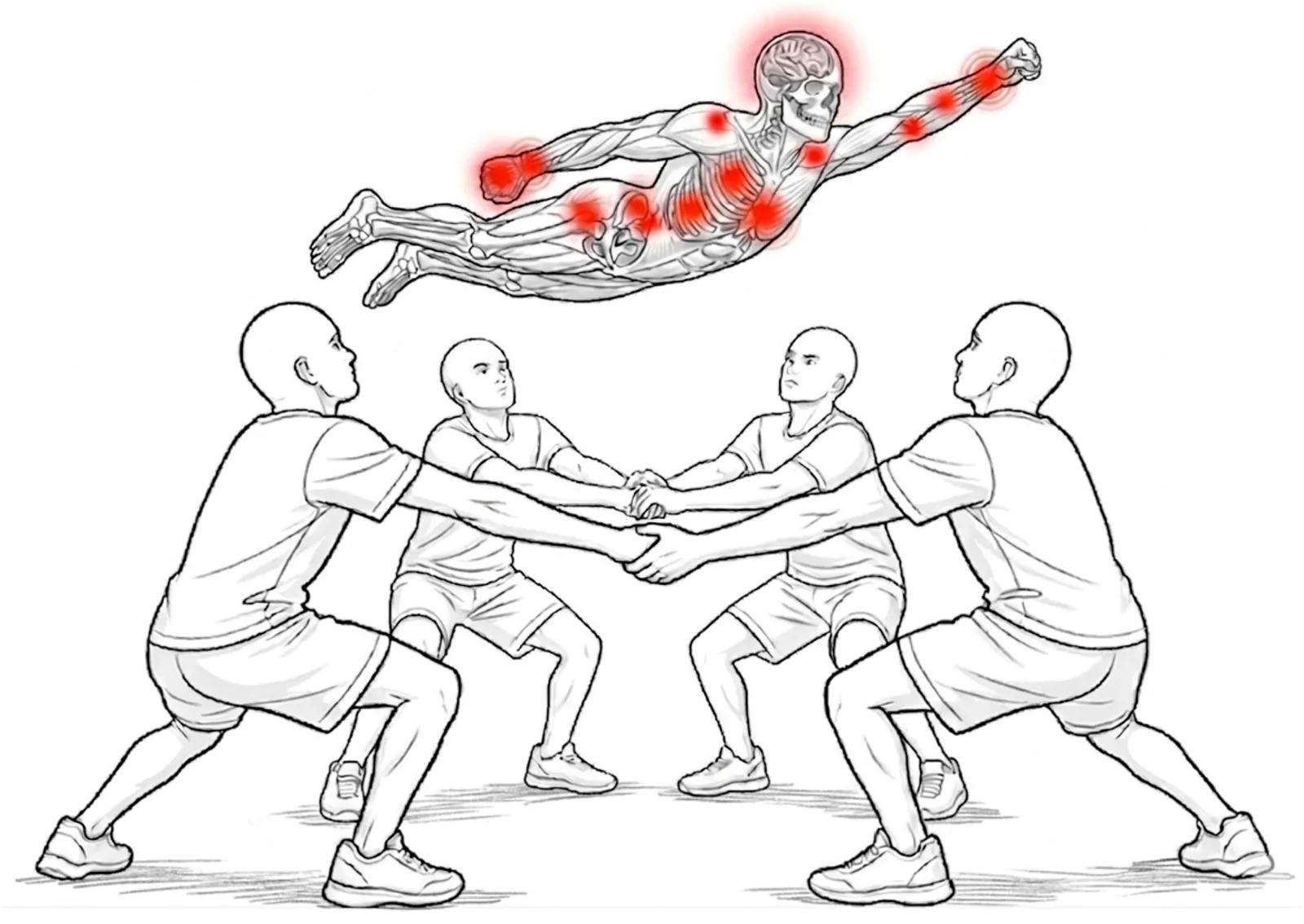
Schematic illustration highlighting the general hazard pattern of a high-impact throw in the characteristic “superman” posture. Red markers indicate primary sites of trauma resulting from a failed catch followed by an uncontrolled fall (height: 1–2 m): distal forearm and wrist fractures due to instinctive protective bracing, head and neck injuries ranging from lacerations to severe traumatic brain injury, and vertebral contusions or spinal compression fractures caused by unattenuated impact forces. The illustration depicts the general hazard pattern described in the broader literature on this challenge and does not represent the injury distribution of the present cohort *(figure created using google gemini)*

While early documented challenges often focused on dangerous ingestions (e.g., the Cinnamon or Tide Pod challenges) or asphyxiation (e.g., the Blackout challenge), there is a growing trend of challenges involving high-risk physical maneuvers [6]. Adolescents are particularly susceptible to participating in these trends due to developmental factors such as increased impulsivity, a reduced capacity for self-regulation, and a high prioritization of peer acceptance [7]. The shift toward video-centric platforms like YouTube and TikTok has further accelerated the visibility and “copycat” replication of these dangerous behaviors. In December 2024, the “Superman challenge” emerged as a particularly hazardous kinetic trend in Europe. In this challenge, a participant is thrown horizontally into the air by peers to capture a “flying” pose; failure to catch the participant results in high-velocity falls and atypical injury patterns Fig. 1 [8]. Despite increasing safety disclaimers and content removal policies by social media platforms, the viral nature of such trends continues to outpace traditional intervention strategies.

The aim of this study is to highlight an unusual cluster of severe forearm injuries resulting from a specific social media trend over a brief period. It underscores the risk of rapid, ‘trend-driven’ spikes in trauma and emphasizes the need for increased clinical awareness regarding changing digital behaviors among young populations.

## Material and methods

We conducted a retrospective, monocentric study at a tertiary pediatric trauma center to evaluate the incidence and patterns of injuries associated with the ‘Superman Challenge’ in December 2024. All patients under 18 years of age were eligible for inclusion.

Clinical and demographic data were extracted from electronic medical records. Injury classification was performed via radiographic evaluation (X-ray) or magnetic resonance imaging (MRI) where indicated. Primary endpoints included:

- Fracture morphology and anatomical location.
- Injury pattern (unilateral vs. bilateral).
- Treatment categorized as cast immobilization or surgical interventions (including reduction with or without K-wire fixation, intramedullary nailing, or arthroscopic wrist stabilization).

Data collection and processing were conducted in accordance with the World Medical Association Declaration of Helsinki. To ensure patient anonymity, all personal identifiers were excluded from the dataset. This study adhered to institutional regulations governing retrospective research involving anonymized clinical data. Following consultation with the Hamburg Ethics Committee, formal ethics approval was waived in accordance with the Hamburg Data Protection Act (*Hamburgisches Datenschutzgesetz*) due to the retrospective and anonymized nature of the study. All patients and parents/legal guardians respectively provided written informed consent for the publication of their data prior to enrollment.

Descriptive statistics were used to summarize patient demographics and injury characteristics. Continuous variables are presented as mean ± standard deviation (SD), while categorical variables are expressed as absolute frequencies and percentages.

## Results

A total of 8 pediatric patients were admitted to our emergency department within a tight 10-day window presenting with acute upper extremity injuries directly sustained during participation in the “Superman Challenge” social media trend. The cohort demonstrated a distinct female predominance, consisting of five female patients (62.5%) and three male patients (37.5%). The mean age of the cohort was 13.25 (±1.03) years, ranging from 12 to 14 years. The observed injury patterns consistently demonstrated a high-energy impact and deceleration mechanism, which contrasts significantly with typical low-impact pediatric falls from standing height.

The structural osseous and soft-tissue injury patterns revealed substantial upper extremity trauma across the cohort. Bilateral distal radius fractures were observed in three patients. Notably, in all bilateral cases, a distinct asymmetric displacement pattern was present, wherein one limb demonstrated significantly greater displacement than the contralateral side. Furthermore, this more severely displaced side consistently presented with a concomitant fracture of the ulnar styloid process (USP), an injury pattern that was also identified in the unilateral cases. Unilateral distal forearm fractures were observed in two patients, which included one Grade I open fracture. One patient experienced a refracture of a pre-existing injury, resulting in the bending and deformation of the in situ elastic stable intramedullary nails. Two patients suffered only contusions. In addition, two of the three cases with bilateral fractures showed accompanying tears of the triangular fibrocartilage complex (TFCC).

**Fig. 2:**
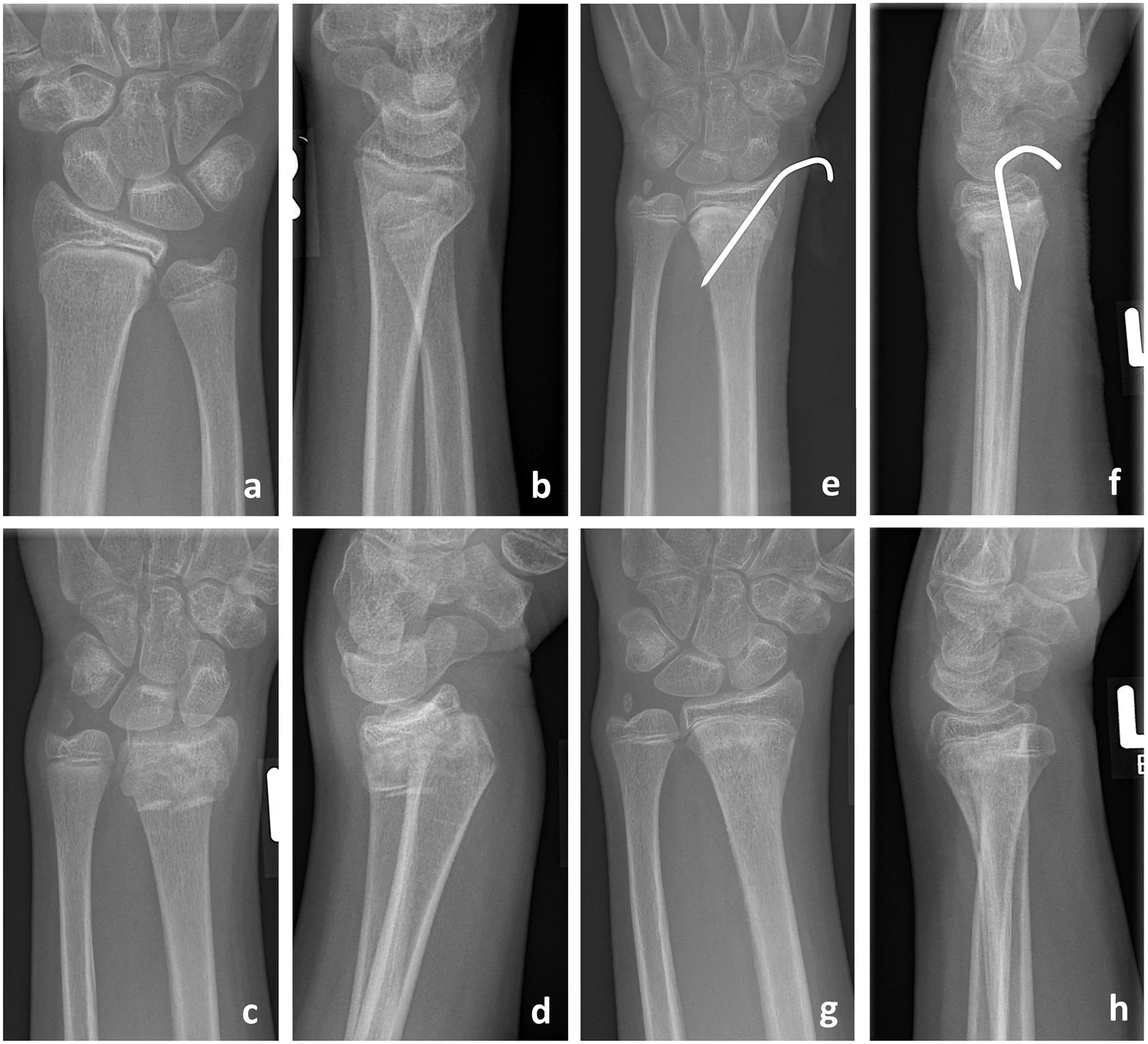
Representative radiographic findings of bilateral distal radius fractures of patient 1 of Table 1 from the “superman challenge” cohort. (**A/B**) Initial radiographs of the right forearm demonstrating a buckle fracture managed conservatively with immobilization. (**C/D**) Initial radiographs of the left forearm showing a dorsally displaced distal radius fracture with avulsion of the USP. (**E/F**) follow-up radiographs of the left forearm 4 weeks after reduction and K-wire osteosynthesis, demonstrating progressive fracture consolidation. (**G/H**) radiographs at 3 months confirming complete osseous healing of the left distal forearm

Therapeutic management was dictated by the degree of displacement and joint stability, where notably, in all bilateral fracture cases, one side showed significantly higher displacement than the other. In all of these cases, the more severely displaced side required closed reduction and, in two cases, an additional percutaneous K-wire osteosynthesis, while the contralateral side was successfully managed with plaster cast immobilization. One of the unilateral fractures was managed successfully with cast immobilization after closed reduction, while the other needed a combined osteosynthesis via K-wire osteosynthesis of the distal radius and ESIN osteosynthesis of the distal ulna. The patient with refracture also required revision surgery with complete hardware exchange. While one TFCC rupture healed under conservative therapy, one required arthroscopic stabilization due to persistent pain and instability. A comprehensive overview of all diagnoses and treatment modalities is provided in Table 1. Radiographs of patient number one from Table 1 with bilateral fractures are presented in Fig. 2.

**Table 1:** Demographic characteristics, treatment patterns, and therapeutic management of the superman challenge case series. Fracture classification using the AO pediatric comprehensive classification of long Bone fractures [9]

Patient	Age /gender	Diagnosis	Management
1	14 female	Left: Dislocated distal meta-epiphyseal radius fracture with avulsion of the USP (23-M/3.1/23u-E/7) and TFCC rupture	Closed reduction and K-wire osteosynthesis and cast immobilization for 6 weeks
		Right: Distal radius buckle fracture (23r-M/2.1)	Cast immobilization for 4 weeks
2	12 female	Right: Dislocated distal radius fracture with avulsion of the USP (23r-E/2.1/23u-E/7) and TFCC rupture	Closed reduction under analgosedation and cast immobilization for 4 weeks Arthroscopic TFCC repair in progress
		Left: Distal non-dislocated radius fracture (23r-E/2.1)	Cast immobilization for 4 weeks
3	12 male	Left: Distal dislocated meta-epiphyseal radius fracture with avulsion of the USP (23r-M/3.1/23u-E/7)	Closed reduction and K-wire osteosynthesis and cast immobilization for 4 weeks
		Right: Distal non-dislocated meta-epiphyseal forearm fracture (23-M/3.1)	Cast immobilization for 4 weeks
4	14 female	Left: I° open displaced distal radius fracturemetaphyseal forearm fracture and avulsion of the USP (23-M/3.1/23u-E/7)	Closed reduction and K-wire osteosynthesis of the distal radius and ESIN osteosynthesis of the distal ulna
5	12 male	bruise and graze wound left hip	Analgesia
6	14 female	bruises on both forearms	Analgesia
7	14 male	Left: Distal dislocated meta-epiphyseal radius fracture with avulsion of the USP (23r-E/3.1/23u-E/7)	Closed reduction and cast immobilization for 4 weeks
8	14 female	Left: Refracture of a forearm shaft fracture (22-D/2.1) previously treated with ESIN osteosynthesis	Revision surgery with complete hardware exchange

## Discussion

Social media has become an integral part of modern life. Children and adolescents, in particular, now spend a significant portion of their leisure time on social networks, blogs, and various forums. Current data indicate that daily screen time exceeds one hour for over 90% of adolescents [2]. While the majority of young people utilize online networks to expand their knowledge regarding education, culture, scientific topics, or reproductive health, there is a growing body of literature reporting negative impacts on mental health. These include cyberbullying, sexting, and the erosion of traditional social boundaries due to the permanent digital availability of content and connections [10, 11]. Social networks now function as primary communication channels that profoundly influence individual lifestyles—either positively through social validation (“likes”) or negatively through their absence.

The combination of intensive use, age-specific impulsivity, and the strong influence of peer groups renders adolescents particularly vulnerable to risky online activities. These factors appear to represent a significant risk for social-media-induced, health-damaging behaviors [7]. In particular, viral “social media challenges” increasingly influence the behavior of children and adolescents, with some trends harboring a substantial risk of injury.

Although risky “dares” are not a new phenomenon, the platform-driven increase in these publicly shared challenges has led to a recent surge in injuries [3]. Several current studies highlight the potential for both psychological and physical harm. However, in the challenges described to date, fractures have been rarely reported. The ‘Superman Challenge’ examined in our study is the first challenge of its kind to have resulted in numerous serious injuries worldwide, including traumatic brain injuries, soft-tissue injuries and various fractures [12–19].

In our case series, we observed an unusual cluster of severe distal forearm fractures that uniformly required surgical intervention. Notably, three of these patients presented with bilateral distal radius fractures. Typically, pediatric bilateral distal radius fractures are rare entities that predominantly affect male patients, result from low-energy trauma, and present as buckle or non-displaced fractures managed conservatively [20]. Conversely, the injuries in our series—sustained during the ‘Superman Challenge’—predominantly affected female patients and were driven by high-velocity trauma.

This high-velocity mechanism is further evidenced by the severe concomitant injuries observed in our cohort. Five of the eight individuals sustained concomitant distal ulna fractures—predominantly avulsion fractures of the USP and two suffered concomitant TFCC tears. While such extensive soft-tissue and osseous injury patterns are characteristic of adult bilateral distal radius fractures following high-energy trauma, they remain exceptionally rare in the pediatric population [21].

Historically, male adolescents have exhibited higher rates of externalizing behavior and impulsive sensation-seeking. However, the digital environment appears to alter these patterns through social and interpersonal pressure factors. Although impulsivity in female adolescents is typically mitigated by a high degree of self-regulatory self-efficacy (SRSE), this protective mechanism can be compromised by ‘fear of social exclusion’. Consequently, the pursuit of group membership can lead to performative risk-taking behavior, effectively narrowing the previously observed gender gap in impulsive behavior, as our case series demonstrates [22, 23].

Furthermore, the acute temporal clustering of these cases is highly remarkable. The presentation of all patients to our emergency department within a narrow two-week window represents a distinctive epidemiological feature; in contrast, viral trends documented in the literature typically exhibit a much more prolonged timeline.

Medical professionals are accustomed to certain seasonal fluctuations in the incidence of specific injuries, such as fractures in ski resorts during winter months [24]. The ‘Superman Challenge’, however, led to a significant increase in injuries out of season. While high-energy pediatric trauma usually correlates with increased outdoor activities during the warmer months from March to October [1], these cases unusually clustered in December. This abrupt surge underscores the challenge as a specific, high-risk injury mechanism that circumvents traditional seasonal trends, resulting in complex fracture patterns that necessitate intensive surgical management.

In the literature, these trends remain insufficiently researched. However, Middelberg et al. recently demonstrated an increase in reports of risky social media challenges [6]. Further research is required to fully understand the underlying mechanisms within social media and to prevent the occurrence of such injuries through targeted intervention.

Nursing and medical staff must be increasingly sensitized to the public health impact of dangerous social media trends. These viral phenomena can precipitate sudden, unpredictable surges in complex and unprecedented injuries. This vulnerability was previously demonstrated by the ‘Plank Challenge’, where planking in unsecured, high-altitude locations resulted in high-energy falls, culminating in severe polytrauma and visceral organ lacerations [25]. Whether trends of this kind could acutely overload emergency departments if undertaken as mass challenges cannot be determined from eight patients, although earlier reports lend plausibility to the concern; for instance, during the COVID-19 pandemic, a sudden influx of severe injuries from the ‘Milk Crate Challenge’ nearly exhausted municipal emergency care resources and healthcare infrastructure in the City of Baltimore [26].

In consequence, this study highlights the importance of parental supervision, school-based education, and regulatory measures by platform operators. Although TikTok has reportedly begun blocking content related to this specific challenge, the widespread availability of similar videos on other platforms highlights existing gaps in the regulation of digital spaces. Possible preventive measures are already being debated at the political level. For instance, Australia recently banned social media use for those under 16 to minimize physical and psychological harm in this age group [27]. Similar calls for stricter age regulation have emerged in other regions, including the European Union. The extent to which such measures will be implemented internationally and whether they will prove effective remains speculative at this time.

## Data Availability

No datasets were generated or analysed during the current study.
